# Potential impacts of polymetallic nodule removal on deep-sea meiofauna

**DOI:** 10.1038/s41598-021-99441-3

**Published:** 2021-10-07

**Authors:** Ellen Pape, Tania Nara Bezerra, Hendrik Gheerardyn, Marius Buydens, Amanda Kieswetter, Ann Vanreusel

**Affiliations:** Marine Biology Research Group, Ghent University, Ghent, Belgium

**Keywords:** Ecology, Biodiversity

## Abstract

Deep seabed mining is potentially imminent in the Clarion Clipperton Fracture Zone (CCFZ; northeast Pacific). Seabed collectors will remove polymetallic nodules and the surrounding surface sediments, both inhabited by meiofauna, along their path. To determine potential impacts of polymetallic nodule removal, we investigated the importance of nodule presence for the abundance, composition and diversity of sediment meiofauna, and evaluated the existence and composition of nodule crevice meiofauna in the Global Sea Mineral Resources (GSR) exploration contract area. Nodule-free and nodule-rich sediments displayed high biodiversity with many singletons and doubletons, potentially representing rare taxa. Nodule presence negatively influenced sediment meiofaunal abundances but did not markedly affect taxonomic composition or diversity. This is the first report on CCFZ nodule crevice meiofauna, whose abundance related positively to nodule dimensions. Though dominated by the same taxa, nodules and sediments differed regarding the taxonomic and trophic composition of the meio- and nematofauna. Nevertheless, there were no taxa endemic to the nodule crevices and nodule crevice meiofauna added only little to total small-scale (~ cm) meiofaunal abundance and diversity. We formulated environmental management recommendations at the contract area and regional (CCFZ) scale related to sampling effort, set-aside preservation and monitoring areas, and potential rehabilitation measures.

## Introduction

The largest, most resource-grade reservoirs of polymetallic nodules are found in the Clarion Clipperton Fracture Zone (CCFZ; Northeast Pacific)^1–4^ and is therefore the most commercially attractive. So far, the International Seabed Authority (ISA), a UN organ with the mandate to regulate mining in the Area (Beyond National Jurisdiction), has assigned sixteen contracts (https://isa.org.jm/minerals/maps, consulted on 23/03/2021) for the exploration of these nodules in the CCFZ. Consequently, sampling effort in the region has risen substantially.

Since commercial interest in mining polymetallic nodules, several studies have addressed the importance of these mineral concretions for the benthic fauna. For CCFZ megafauna, the largest benthic organisms typically > 1 cm, previous research showed elevated abundances and a distinct community composition in nodule-bearing *vs.* (virtually) nodule-free sediments^5–7^. For macrofauna (> 250–300 µm) the effect of nodule presence appears ambiguous (see also Washburn et al.^8^) as studies have demonstrated a positive^9^, negative^10^ or no marked influence^11^. Meiofauna (> 32 µm) research in several eastern CCFZ localities^12–15^ showed reduced abundances in nodule-bearing sediments, presumably governed by the lower sediment (substrate) availability. Regarding meiofaunal compositional differences between sediments with and without nodules, Singh et al.^16^ reported no significant differences in nematode genus composition, whilst significant dissimilarities were observed for copepod^15^ and nematode^14^ species in the Institut Français de Recherche pour l’Exploitation de la MER (IFREMER) contract area in the CCFZ.

Decades ago, researchers discovered meiofauna inside the sediment-filled crevices of nodules from the Peru Basin, which they termed “crevice meiofauna”^17,18^. This crevice meiofauna was dominated by nematodes, of which the composition diverged from that in the surrounding sediments^17^. To date, no studies have been published on nodule crevice meiofauna from the CCFZ.

Nodule collectors will not only remove polymetallic nodules but are also expected to remove (though sediments may be redeposited or moved elsewhere) and compact the surface layer of the ambient soft sediments^19–21^ along its track, both of which are inhabited by meiofauna. This habitat removal or disturbance will inevitably result in the localized (i.e. in the path of the nodule collector) loss of meiofaunal abundance and biodiversity. Impacts at larger spatial scales should be mitigated where possible through effective environmental management^22^. Parts of future exploitation contract areas will not be mined, either because of technical unfeasibility^19^, economical unattractiveness (insufficient nodules)^23^, or because these will be set aside as a preservation reference zone to monitor mining impacts^24^ and/or as areas specifically aimed at environmental protection (GSR, pers. comm.). These areas, if unaffected by mining, may aid to safeguard biodiversity if these can (help to) sustain viable populations of most of the taxa present in the mining areas^24^. Eventually, these areas may serve as sources for recruits for the recolonization of mining areas^25^.

To evaluate potential deep seabed mining impacts on meiofauna, we investigated to what extent the presence of polymetallic nodules affects their abundance, composition and diversity. To this end, analyses of samples from the Global Sea Mineral Resources (GSR) contract area in the eastern CCFZ were threefold. Firstly, we tested whether sediment meiofaunal abundance, composition and diversity differed consistently between a (naturally) nodule-free station and two nodule-rich stations. Additionally, we checked for relationships between these meiofauna community attributes and a suite of sediment environmental variables indicative of food availability (pigment concentrations, content of nitrogen and organic carbon) and physical habitat characteristics (granulometry). Secondly, we compared the nodule crevice meiofauna with the sediment meiofauna in terms of abundance, composition and diversity. Finally, the contribution of the crevice meiofauna to total small-scale (~ cm, at the scale of a MUC core) meiofaunal abundance and diversity was quantified.

## Results

### Nodule-free *vs.* nodule-rich sediments

#### Meiofaunal abundance and higher taxon composition

Meiofaunal abundances differed between the three stations (PERMANOVA, F = 8.32, P = 0.003), with NodFree (126.8 ± 29.0 ind. 10 cm^−2^) harboring more meiofauna than the nodule-rich stations (NodRich_A: 87.0 ± 21.0 ind. 10 cm^−2^, NodRich_B: 45.4 ± 31.5 ind. 10 cm^−2^). Pairwise tests showed this difference was significant between NodFree and NodRich_B (F = 14.48, P = 0.03), but not between NodFree and NodRich_A (F = 4.00, P = 0.06), though the P-value was close to 0.05. Differences between stations were largely, yet not entirely, caused by the differential sediment (i.e. substrate) availability (Nodfree: 368.4 ± 101.9 ml, NodRich_A: 264.2 ± 20.9 ml, NodRich_B: 219.2 ± 31.5 ml) since the difference between stations was much less significant (PERMANOVA, F = 5.51, P = 0.03) upon including sediment volume as a covariate (PERMANOVA, F = 4.31, P = 0.07). None of the Spearman–Rank correlations between meiofauna abundance and the environmental variables measured were significant (all P ≥ 0.05).

Meiofauna higher taxon composition was comparable between stations (Fig. 2a), dominated by nematodes (89–95%), copepods (4–9%) and nauplii (0.4–3.4%). Other taxa encountered were: Bivalvia, Gastropoda, Gastrotricha, Halacaroidea, Kinorhyncha, Ostracoda, Polychaeta, Tanaidacea, Tantulocarida and Tardigrada. There was no significant correlation between meiofauna taxon composition and any of the environmental variables (Supplementary Table S1).

#### Nematode community composition and diversity

**Figure 1:**
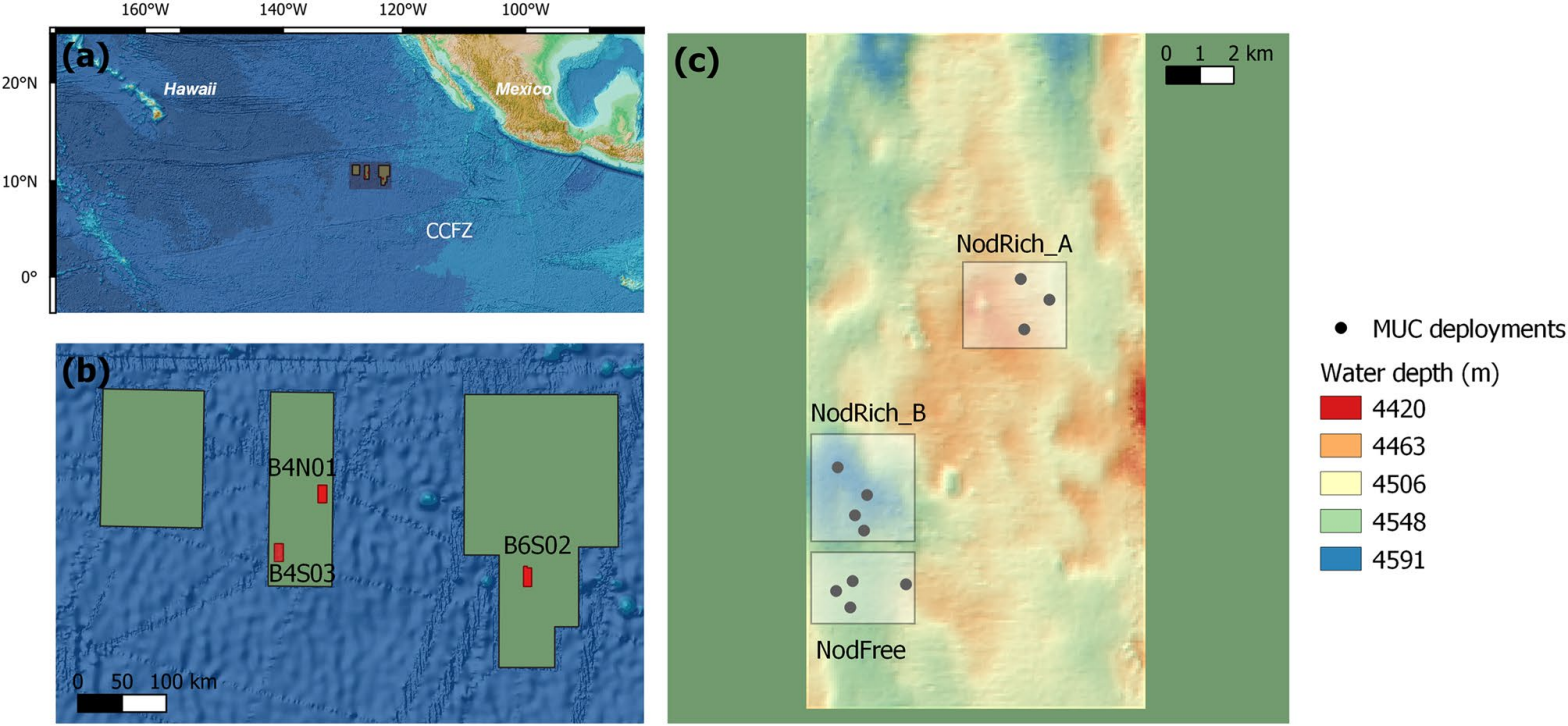
Maps of the sites and stations sampled. Shown are (**a**) the CCFZ with an indication of the GSR contract area, (**b**) the GSR contract area with the different sites sampled, (**c**) the nodule-free (NodFree) and nodule-rich (NodRich_A and NodRich_B) stations at site B4S03.

**Figure 2:**
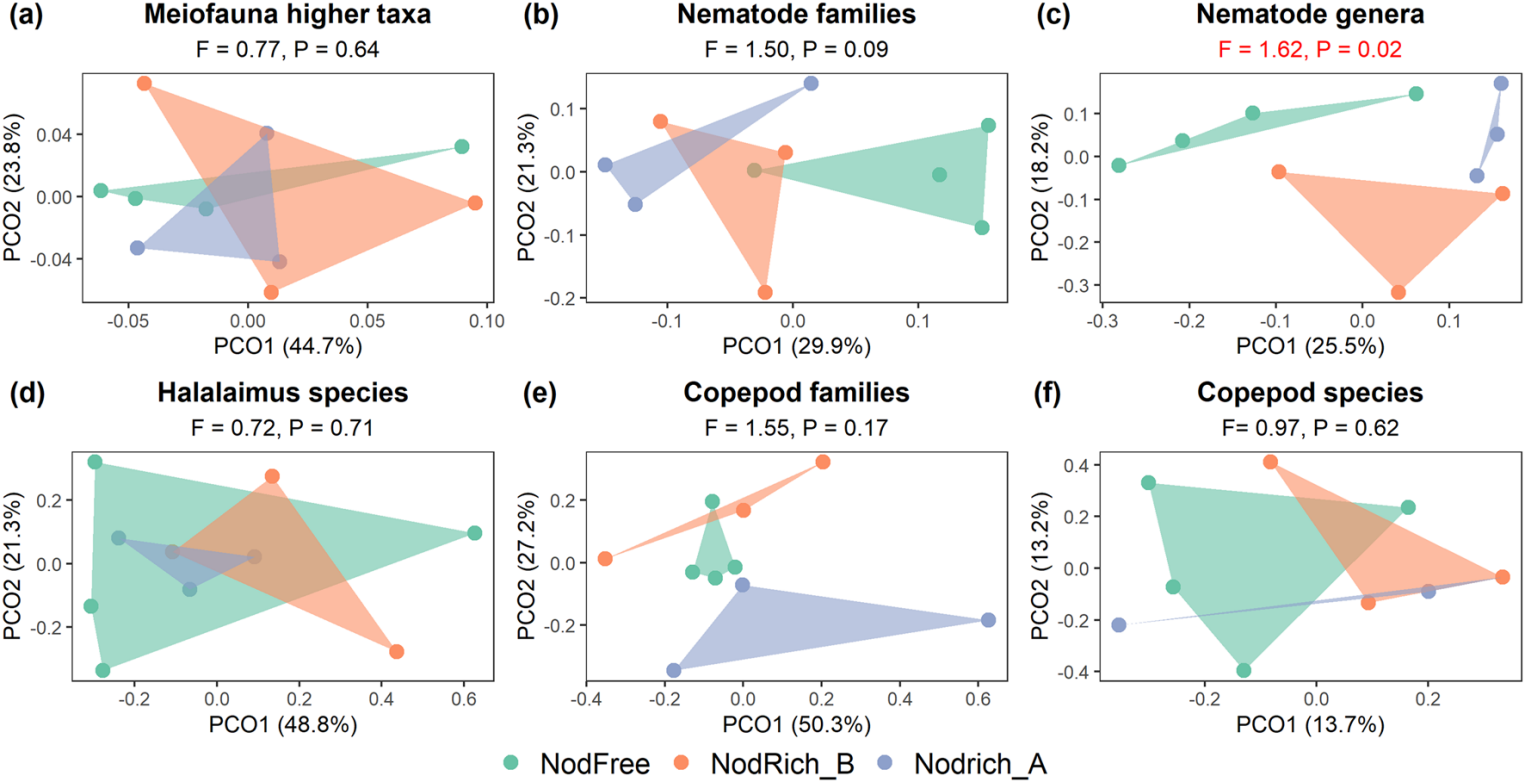
PcoA plots comparing taxonomic composition between the nodule-free (NodFree) and nodule-rich (NodRich_A and NodRich_B) stations (0–5 cm sediment depth). (**a**) Meiofauna higher taxa, (**b**) nematode families and (**c**) nematode genera, (**d**) *Halalaimus* species and copepod (**e**) families and (**f**) species. Convex hulls are drawn around samples from the same station. The F and P-values for the PERMANOVA tests are shown on top; for statistically significant tests, these are indicated in red.

We identified 1093 nematodes belonging to 27 families and 73 genera. Forty-three percent of the nematode genera were singletons (19) or doubletons (12). The three stations displayed a similar family composition [Fig. 2b and Supplementary Fig. S1a; dominated by Monhysteridae (14–46%), Chromadoridae (10–31%) and Xyalidae (5–17%)], but a significantly different genus composition (Fig. 2c). Nevertheless, there was no clear-cut effect of nodule presence on genus composition, since (1) the nodule-rich samples did not group together and separately from the nodule-free samples in the PCoA plot (Fig. 2c), and (2) pairwise PERMANOVA tests revealed a borderline significant difference only between NodFree and NodRich_A (F = 1.85, P = 0.05), which were also furthest apart (see Fig. 1b). In each core, *Monhystrella/Thalassomonhystera* and *Acantholaimus* prevailed (Supplementary Fig. S1b), and compositional differences between stations were mainly driven by the less abundant genera. Neither nematode family nor genus composition were related significantly to any of the environmental variables (Supplementary Table S1). The differential nematode genus composition between stations was not accompanied by a different trophic composition (PERMANOVA, F = 1.12, P = 0.40; Supplementary Figure S2).

**Figure 3:**
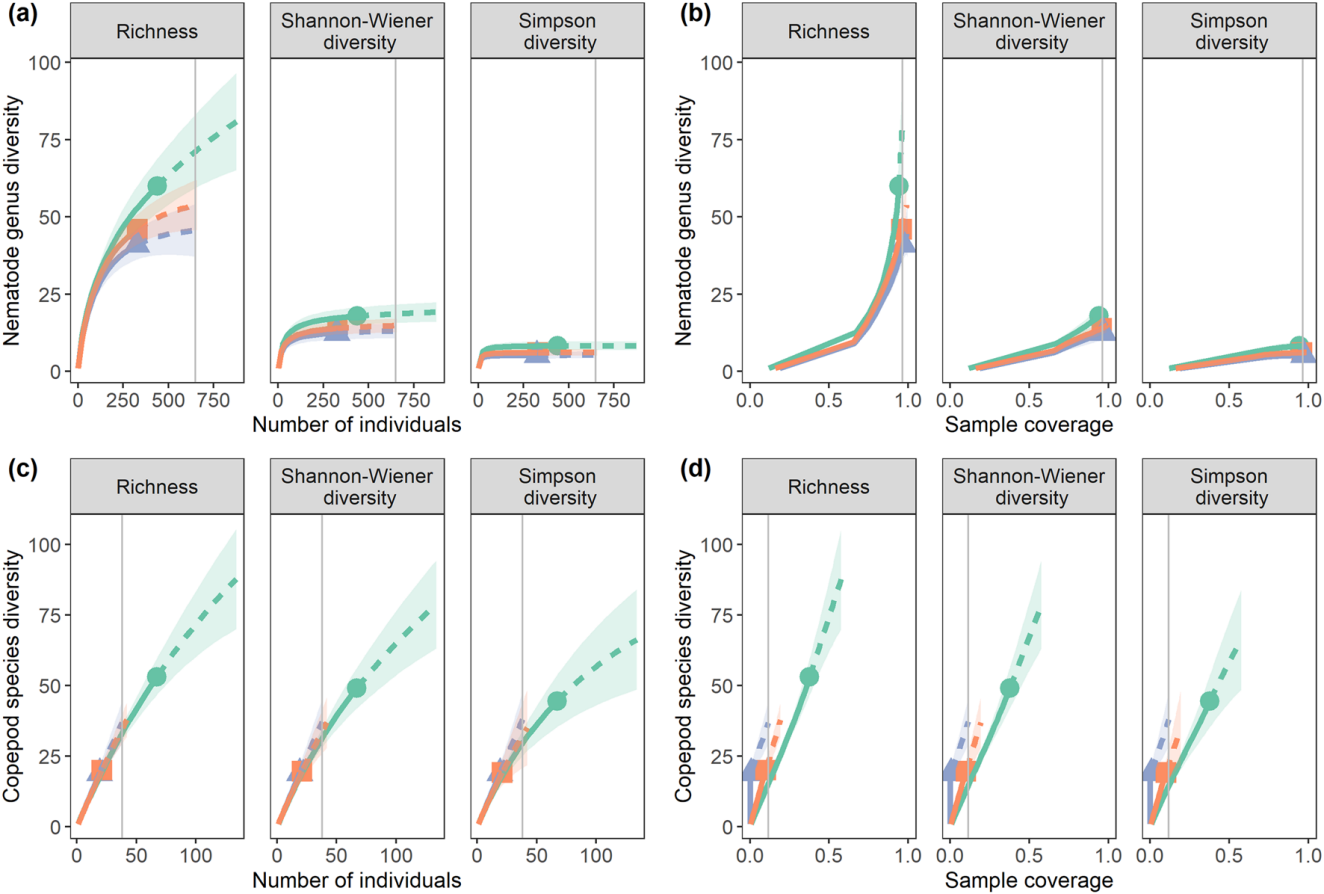
Nematode and copepod diversity at the nodule-free (NodFree) and nodule-rich (NodRich_A and NodRich_B) stations. Shown are sample-size (left) and sample coverage-based (right) rarefaction (interpolated) and extrapolation (predicted, guided by asymptotic estimators) curves for (**a**,**b**) nematode genus and (**c**,**d**) copepod species diversity, based on abundances. The different panels show Hill numbers of orders (q) 0 (Richness), 1 (Shannon–Wiener diversity) and 2 (Simpson diversity). The vertical grey line denotes the base sample size (left) or the base sample coverage (right). Shaded areas represent 95% confidence intervals.

Sample coverage for assessing nematode genus richness exceeded 94%. Sample-size- and coverage-based R/E curves always displayed the same diversity ordering, i.e. NodFree > NodRich_B > Nodrich_A (Fig. 3a,b). Significant differences between NodFree and NodRich_A for genus richness were implied by the non-overlapping 95% confidence intervals at the base sample size and coverage. Dissimilarities between stations declined with increasing order of q (and thus declining sensitivity to sample size) for both R/E plots.

**Figure 4:**
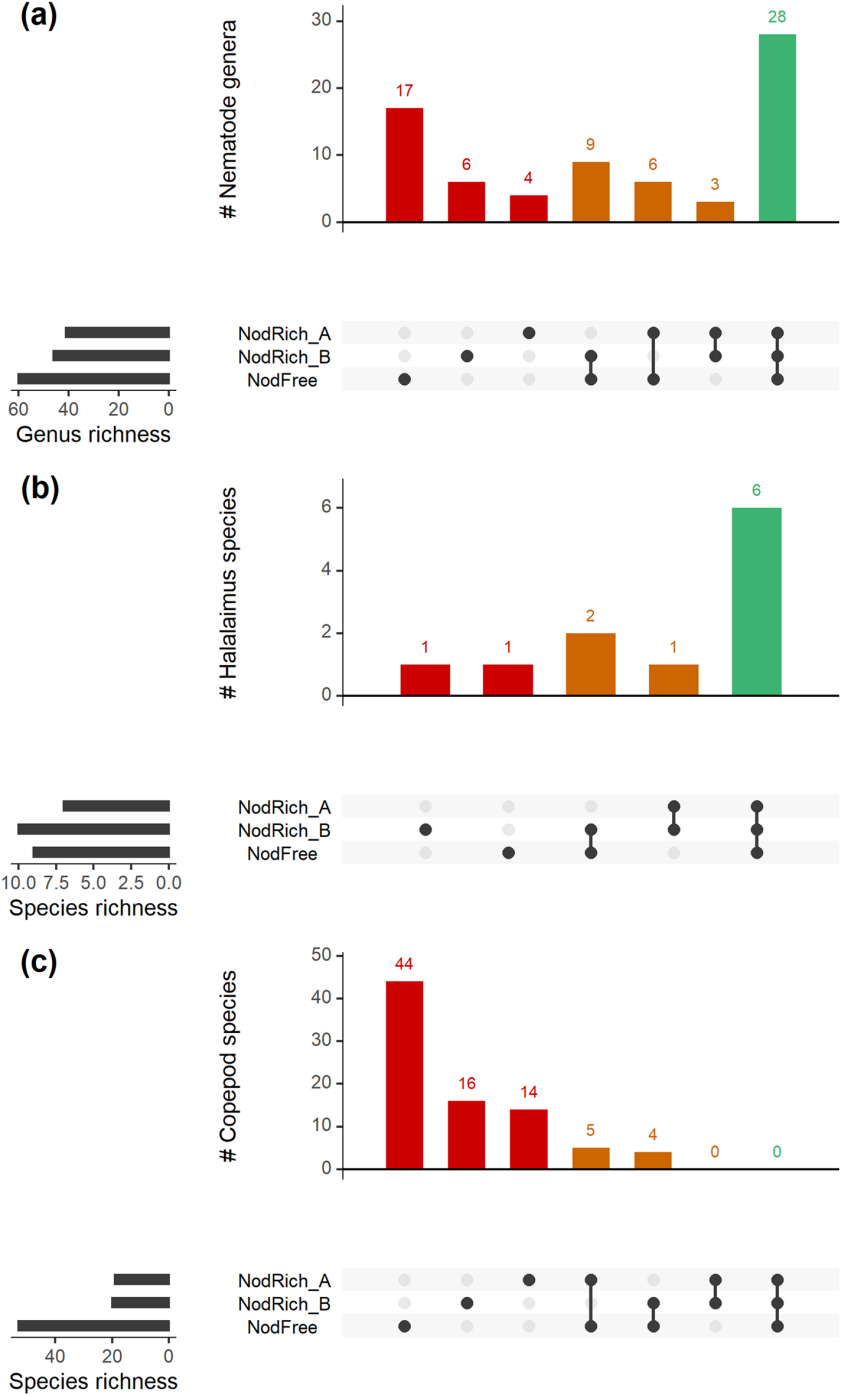
UpSet plots for the nodule-free (NodFree) and nodule-rich stations (NodRich_A and NodRich_B) showing the number of shared and unique taxa. (**a**) Nematode genera, (**b**) *Halalaimus* species and (**c**) copepod species. The color of the bars denotes the commonness of taxa, with red: unique taxa, yellow: taxa shared between two stations, green: taxa shared between all three stations.

Besides highest genus richness, NodFree also displayed the highest number of unique genera (Fig. 4a). The UpSet plot (Fig. 4a) further disclosed that 28 genera (out of 74, or 38%) occurred at all stations, and that three, relatively rare, genera occurred in both NodRich stations (i.e.* Prochromadorella, Perspiria, Ceramonema*), but not at NodFree.

*Halalaimus* identification yielded eleven species (65 individuals in total, 1–10 per core) (Supplementary Figure S1c). Owing to the high between-sample variability, no significant difference in species composition between stations emerged (Fig. 2d). Significant links between *Halalaimus* species composition and any of the environmental variables were absent (Supplementary Table S1).

Sample coverage for assessing *Halalaimus* species diversity was ≥ 84%. The sample-size and coverage-based R/E curves exhibited the same diversity ranking with NodRich_B > NodFree > NodRich_A for all Hill numbers at the given base sample size and coverage (Supplementary Fig. S3). However, since the confidence bands of both types of R/E curves for the three stations strongly intersected, no conclusion was possible regarding the statistical significance of these differences. As for nematode genus diversity, the distinction between stations lessened with decreasing sensitivity to sample size (increasing orders of q). The UpSet plot (Fig. 4b) revealed that more than half of the *Halalaimus* species occurred at all stations.

#### Copepod community composition and diversity

In total, 107 copepods were identified to lower taxonomical level, yielding thirteen families and 104 species. Seventy-seven species (75%) were rare being either singletons (n = 65, 63%) or doubletons (n = 12, 12%). Neither family nor species composition differed significantly between stations (Fig. 2e,f). No significant correlations existed between any of the environmental variables and either family or species composition (Supplementary Table S1).

For the assessment of copepod species richness, sampling effort was insufficient as coverage was maximally 38% (for NodFree). The sample-size based R/E curves for the three Hill numbers coincided for the three stations, indicating comparable diversity (Fig. 3c). In contrast, the sample-coverage based curves (Fig. 3d) and their 95% confidence intervals were more segregated, and curves were consistently ranked as follows: NodRich_A > NodRich_B > NodFree. For species richness (q = 0), the confidence intervals associated with the station curves did not overlap suggesting significant differences. For q > 0, only the confidence bands of the NodRich_A and NodFree curves were separated. No copepod species was found at all stations, and 89% was confined to one (Fig. 4c). Especially the NodFree station, with the highest observed species richness, hosted many unique species.

### Nodule crevices *vs.* sediments

#### Meiofaunal abundance and higher taxon composition

**Figure 5:**
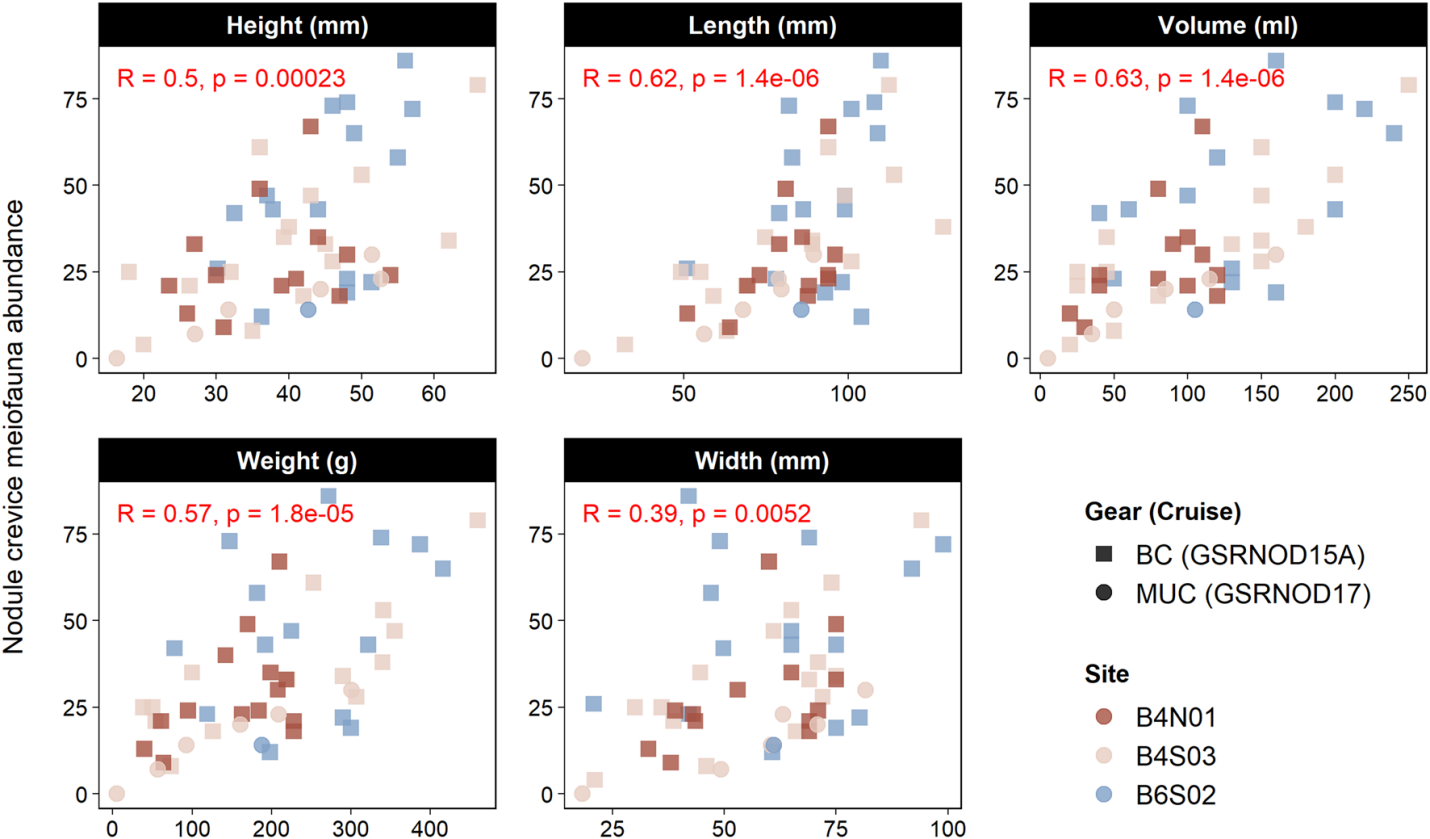
Nodule crevice meiofaunal abundance in function of physical nodule characteristics. Colors represent sites, and shapes denote sampling gear (*BC* boxcorer, *MUC* multiple corer) and expedition. The Spearman rank correlation coefficient (R) and the associated P-value are given in red for each physical nodule characteristic.

The crevices of the polymetallic nodules from the GSR contract area harbored between 0 and 86 meiofaunal individuals (median = 29). All physical nodule characteristics related positively to crevice meiofaunal abundance (Fig. 5).

Overall, we found 15 higher meiofaunal taxa; eight were shared between nodule crevices and sediments, whilst seven rare taxa were unique to the sediments (Supplementary Fig. S4). The crevice meiofauna of most nodules were dominated by nematodes (median relative abundance: 85%) and copepods (12%), similar to sediment meiofauna.

**Figure 6:**
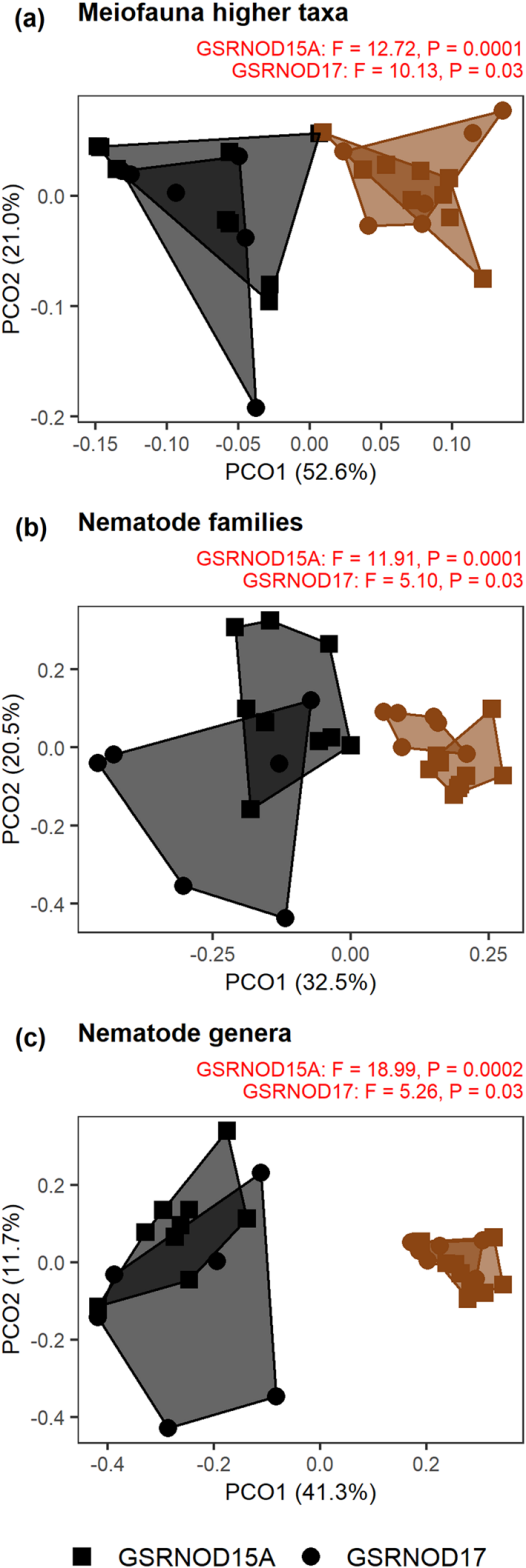
PcoA plots comparing taxonomic composition between nodule crevices (black) and sediments (brown) sampled during GSRNOD15A (squares) and GSRNOD17 (circles). Plotted are (**a**) higher meiofauna taxa, (**b**) nematode families and (**c**) nematode genera. Convex hulls are drawn around samples from the same substrate and expedition. The F and P-values for the PERMANOVA tests are shown on top of each plot; as all tests were significant, these are all indicated in red.

Significant differences were observed between nodule crevices and sediments in higher taxon composition (Fig. 6a). The nodule crevice meiofauna was a subset of the sediment meiofauna (Supplementary Fig. S4), as the spatial turnover component of beta diversity, i.e. the difference in taxon composition between nodules and sediments, amounted to zero. Nauplii, which were less abundant inside the nodules, contributed most to this dissimilarity between substrates (simper, GSRNOD15A: P = 0.0003, GSRNOD17: P = 0.03). There was no distinction in composition between the three sites sampled during GSRNOD15A (PERMANOVA, F = 1.16, P = 0.33).

#### Nematode community composition and diversity

Overall, 32 nematode families were identified, of which most (72%) were shared between the two substrates (Supplementary Fig. S5a). Six families (i.e. Benthimermithidae, Ceramonematidae, Draconematidae, Leptosomatidae, Neotonchidae and Meyliidae) were restricted to the sediments, and three (Axonolaimidae, Ethmolaimidae, Thoracostomopsidae) to the nodule crevices. One hundred and twelve genera (see Supplementary Fig. S5b) were identified, of which half (n = 58, 52%) occurred in both substrates. Twenty-seven percent was only encountered in the sediments whilst 21% was unique to the nodules.

Similar to the sediments, monhysterids were the predominant family in the crevices of the nodules sampled during GSRNOD15A (median relative abundance: 41%) and GSRNOD17 (25%) (Supplementary Fig. S6). The identity of the other dominant families in the nodule crevices differed between expeditions. The second most abundant families in the GSRNOD15A nodule crevices were the Chromadoridae (14%) and Xyalidae (12%), which also dominated the sediments. In contrast, Camacolaimidae (13%) and Rhabdolaimidae (7%) were the second most abundant in the GSRNOD17 nodule crevice samples, but these were rare in the sediments (Supplementary Fig. S6). Despite the shared dominant family, sediments and nodule crevices displayed a significantly different family composition (Fig. 6b). The families that contributed most and significantly to this difference according to the simper analysis were either absent from the nodule samples (GSRNOD17, Desmodoridae: P = 0.01) or found in only one nodule crevice sample (GSRNOD15A, Desmoscolecidae: P = 0.0001; GSRNOD17, Microlaimidae: P = 0.03, Xyalidae: P = 0.03).

Nodule crevice samples had a more variable family composition than sediment samples (GSRNOD15A: PERMDISP, F = 14.55, P < 0.0001, GSRNOD17: F = 12.43, P = 0.05; Fig. 6b). The three sites targeted in 2015 showed a comparable family composition (PERMANOVA, F = 1.46, P = 0.18).

Also at genus level, nodule crevices and sediments showed a significantly different composition (Fig. 6c), though *Monhystrella/Thalassomonhystera* (median relative abundance, GSRNOD15A: 50%, GSRNOD17: 38%) dominated both substrates (Supplementary Fig. S7). *Deontolaimus*, which was rare in the sediments (GSRNOD15A: 2%, GSRNOD17: 0.9%), was the second most abundant genus (GSRNOD15A: 17%, GSRNOD17: 14%) in the nodule crevices. This genus contributed most to the difference between substrates (simper, GSRNOD15A: P = 0.007, GSRNOD17: P = 0.09), although for the GSRNOD17 samples its contribution was insignificant. Interestingly, the distinction between the two substrates increased with decreasing taxonomical level (Fig. 6). Both nestedness (59 ± 24%) and turnover (41 ± 24%) contributed to the difference in genus composition between substrates. Sites B6S02, B4S03 and B4N01 showed a comparable genus composition (PERMANOVA, F = 1.70, P = 0.14).

Nematode trophic composition differed significantly between nodule crevices and sediments sampled during GSRNOD15A (PERMANOVA, F = 38.40, P = 0.0001; Supplementary Fig. S8). The simper analysis revealed significant differences for three of the four trophic groups: 2B (P = 0.0002; median relative abundance of 2% and 10% in sediments and nodule crevices, respectively) and 1B (P = 0.0002; 35% and 42% in sediments and nodule crevices, respectively) were relatively more abundant inside the nodules, whilst 1A attained higher relative abundances in the sediments (P = 0.005; 31% and 8% in sediments and nodule crevices, respectively). For the GSRNOD17 samples, trophic composition was similar between the two substrates (PERMANOVA, F = 1.91, P = 0.06). Nodule crevices were more variable in terms of nematode trophic composition than the sediments sampled during GSRNOD17 (PERMDISP, F = 1.91, P = 0.02).

**Figure 7:**
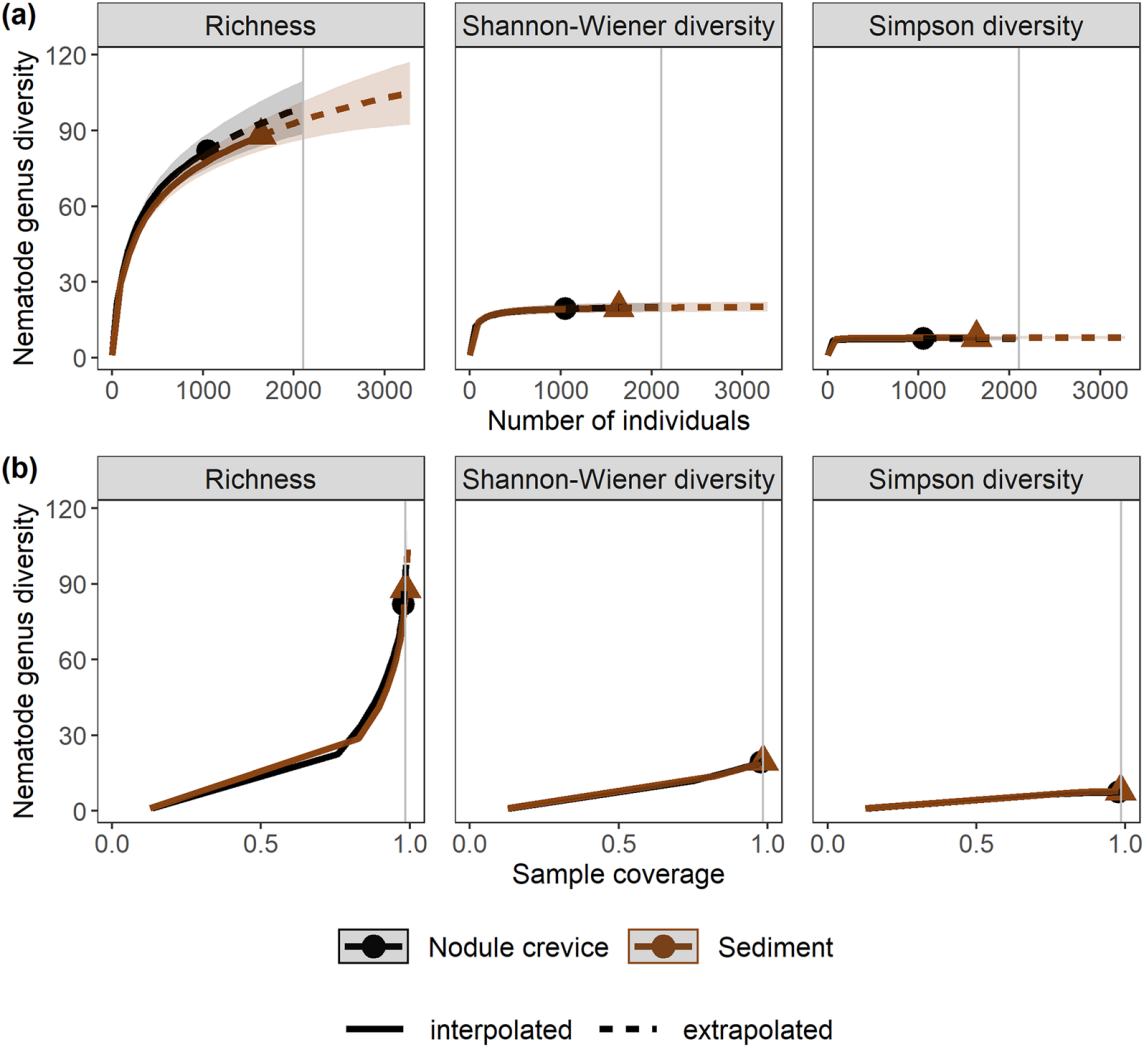
Nematode genus diversity in nodule crevice and sediment samples. Shown are (**a**) sample-size- and (**b**) sample coverage-based rarefaction (interpolated) and extrapolation (predicted, guided by asymptotic estimators) curves for nematode genus diversity based on abundances. The different panels show Hill numbers of orders (q) 0 (Richness), 1 (Shannon–Wiener diversity), and 2 (Simpson diversity). The vertical grey line denotes the base sample size in (**a**) and the base sample coverage in (**b**). Shaded areas represent 95% confidence intervals.

Nematode genus diversity was comparable between nodule crevices and sediments given the same sample size (number of individuals) and sample coverage (Fig. 7). All 14 *Halalaimus* species identified occurred in the sediments; half were found in the nodule crevices too. Seven species were not observed in the nodule crevice samples, including *H. abyssus*, one of the predominant *Halalaimus* species in the sediments.

### Contribution of nodules to meiofaunal abundance and diversity

Based on the (6) MUC cores sampled during GSRNOD17 that contained nodules, nodules added on average 5% to total meiofaunal abundance in a core. Since all of the meiofaunal higher taxa in the nodule crevices were also present in the sediments, the nodules did not add to any of the taxon diversity indices. For nematode genus diversity, there was an increase owing to the presence of nodules of 9% (71 → 77) for genus richness, 6% (54 → 57) for Shannon diversity, and 7% (45 → 48) for Simpson diversity.

## Discussion

### Sediment meiofaunal diversity in the GSR contract area

Local meio- and macrofaunal diversity within deep-sea sediments may rival or even exceed levels in shallow waters^26–28^, and is generally characterized by the presence of many taxa represented by few individuals^29^. This high biodiversity and the occurrence of many rare taxa has been documented also within the CCFZ for different benthic size groups at different localities^11,12,14,30–35^. The present meiofauna dataset from the GSR contract area confirms this pattern, revealing high alpha (local; i.e. within a station) nematode genus and copepod species diversity with 43% and 75% rare genera and species, respectively. Copepods were extremely speciose with nearly every individual belonging to a different species. Similarly high copepod species diversity was reported for the CCFZ IFREMER contract area^15^ but also other abyssal regions^37,38^. It should be noted that nematode diversity indices were calculated based on subsets of specimens which can lead to underestimations. However, we expect any underestimations to be confined mainly to taxon richness which is strongly dependent upon sample size^36^.

Rare taxa may fulfill unique functions^39^ or confer functional redundancy^40^ and thus ultimately contribute to higher ecosystem functioning. Nevertheless, the question remains whether the high proportion of singletons and doubletons, as observed in most abyssal studies including ours, are truly representative of rare taxa or whether these are the result of under sampling^29,41^. Increasing sampling effort could provide a more comprehensive overview to the degree and spatial scale (local, regional or global) of rarity, i.e. spatial variability in abundance and the size of distribution ranges^29,42^. As (locally, regionally or globally) rare species are more prone to (local, regional or global) extinctions, such information will ultimately contribute to spatial management plans for mining areas. The high fraction of singletons and doubletons was not the only finding suggesting that meiofauna was undersampled in this study. Firstly, the low number of samples led to few possible permutations for some statistical tests and thus low statistical power at the chosen significance level (e.g. pairwise tests for comparison of nematode genus composition between stations)^43^. Secondly, sample coverage for the assessment of copepod species diversity in the study area was very poor, and sampling effort should be at least doubled to obtain reliable copepod species diversity data.

### Nodule-free *vs.* nodule-rich sediment meiofauna

Future nodule collection will remove both nodules and the surrounding surficial sediments. Therefore, we investigated if and to what extent meiofauna communities differ between nodule-bearing sediments, and those naturally devoid of nodules, which will not be exploited but which may be impacted indirectly by sediment plumes generated by seabed mining activity^44^. Consistent with previous CCFZ studies^12,14,15^, the nodule-free sediments in the GSR contract area harbored more meiofauna per surface area than the nodule-rich sediments, mainly owing to the higher substrate availability (higher sediment volume per surface area) in the former environment. In accordance with previous research^14–16^, both environments were dominated by the same meiofaunal (i.e. nematodes and copepods) and nematode taxa (i.e.* Monhystrella/Thalassomonhystera* and *Acantholaimus*). No marked effect of nodule presence on nematode genus composition was observed, consistent with Singh et al.^16^ and Miljutina et al.^14^. This was also concluded for macrofauna from the same stations in the GSR contract area^11^. We did detect differences in nematode genus composition between the three stations, caused by the less abundant genera. A similar observation was noted in Glover et al.^45^ where the same group of abundant polychaete species dominated different CCFZ sites and any compositional differences were mainly governed by rare species. *Halalaimus* nematode species did not differ significantly in composition between stations and thus seemed to be unaffected by nodule presence. However, a significant effect of nodule presence on species composition was reported for the IFREMER contract area^14^, where all nematodes were identified to species. Due to the high within-station variability, significant differences between stations, and thus between nodule-free and nodule-rich sediments, were not found for copepod species. In contrast, Mahatma^15^ documented a significantly different copepod species composition between nodule-free and nodule-rich sediments in the IFREMER contract area. This disagreement may partly be related to the higher number of replicate samples collected in both environments (here: 3–4, Mahatma^15^: 5) and consequently the higher number of individuals identified (here: 107, Mahatma^15^: 424) by Mahatma^15^.

The present dataset did not reveal marked and consistent differences in nematode genus or copepod species diversity between nodule-free and nodule-rich sediments. Comparable species diversity in nodule-free and nodule-bearing sediments was observed for macrofauna in the GSR contract area^11^ and copepods in the IFREMER contract area^15^. In the present study, higher abundance in nodule-free sediments was accompanied by higher nematode genus richness, though differences between nodule-free and nodule-rich stations were smaller for diversity indices which are less sample size-dependent. No inferences could be made about the effect of nodule presence on nematode species diversity in the GSR contract area, but in the IFREMER contract area nematode species diversity was comparable between nodule-free and nodule-bearing sediments^14^.

Like Hauquier et al.^12^, we observed no significant relationships between meiofaunal community attributes and sediment environmental parameters indicating food availability (pigment concentrations, content of nitrogen and organic carbon) and physical habitat characteristics (granulometry). Meiofaunal abundance and community composition are determined by a complex interplay of biotic and abiotic factors that act simultaneously at different spatial scales^46^. Therefore, the simple correlation test employed here for abiotic factors may not have captured this adequately. Additional factors, like biotic interactions^46^, may also be important in shaping meiofauna communities. Further, environmental and meiofaunal variables were measured on separate MUC cores and thus the high variability at local scale (i.e. between cores of the same MUC deployment) as shown by others^46,47^, may be responsible for the lack of significant relationships.

### Nodule crevice *vs.* sediment meiofauna

Decades ago meiofauna was discovered inside the crevices of nodules from the Peru Basin^17,18^. Yet, this is the first study to evidence the existence of this so-called nodule crevice meiofauna in the CCFZ. The Peru Basin nodules harbored more meiofauna (Thiel et al.^17^: max. 170 ind. per nodule, Bussau et al.^18^: 112 ind. per nodule) than those from the GSR contract area (max. 86 ind. per nodule). This may be related to the larger size of the Peru Basin nodules (Thiel et al.^17^: 10–16 cm diameter, here: 3–12 cm length, see Fig. 5), as evidenced here by the positive correlation between nodule dimensions and crevice meiofauna abundance.

As documented for the Peru Basin^18^, nodule crevices and sediments from the GSR contract area were dominated by the same meiofaunal (nematodes and copepods) and nematode taxa (*Monhystrella/Thalassomonhystera)*. In agreement with the Peru Basin nodule crevice meiofauna studies^17,18^, the two substrates were inhabited by distinct meiofaunal and nematode communities. For the meiofauna taxa, only a subset of the taxa which were relatively abundant in the sediment, were also present inside the nodules. However, for nematode genera, turnover contributed substantially to the difference in composition between substrates, with roughly 25% of the genera being unique to either sediments or nodules. Importantly, all nematode genera found in the nodule crevices but not the sediments were all reported before from other sediment locations; hence, there were no genera endemic to the nodule crevices. The dissimilar composition between sediments and nodule crevices was largely driven by the nematode genus *Deontolaimus*, which was rare in the sediments but relatively abundant in the nodule crevices; this was also observed by Bussau et al.^18^. Similarly, some of the *Halalaimus* species abundant in the sediments were unable to enter or survive in the nodules. Given the increasing divergence between nodule crevice and sediment communities with decreasing taxonomical level, it is expected that the two substrates differ even more regarding species composition. The difference in nematode genus composition between substrates was translated into a different trophic composition based on buccal morphology^48^, implying potentially different nematode feeding strategies inside *vs.* outside the nodules (but see^49–51^). Nematodes of trophic group 2B may be able to use their large teeth to scrape off the bacteria^52,53^ or Foraminifera^54^ from the nodule crevice walls, but this hypothesis merits further study.

The sediments of the environmentally similar GSR sites B4S03, B4N01 and B6S02 were shown before to harbor comparable meiofaunal communities^55^, and here we observed the same for the nodule crevices. Some comparisons between nodules and sediments revealed slightly different results for the two expeditions; potential reasons include the high spatio-temporal variability evidenced for other CCFZ localities^56^, the different sampling gears (with the MUC samples from GSRNOD17 containing smaller nodules, see Fig. 5) and the large difference in sampling effort.

### Nodule crevice *plus* sediment meiofauna

We found a limited increase in small-scale (i.e. at the scale of a MUC core) meiofaunal diversity and abundance owing to the fauna in the nodule crevices. Although based on six cores only, this finding can likely be generalized since crevice meiofauna abundance was overall low. Nonetheless, further investigation on the effect of nodule size (shown to correlate with meiofauna crevice abundance) and nodule facies^57,58^ is necessary to fully comprehend their importance for total small-scale meiofauna diversity and abundance. Importantly, this study evaluated nematode genus diversity and found that nodules may be a more important contributor to total species-level diversity. This is implied by the increasing divergence in community composition between nodule crevices and sediments with increasing taxonomical resolution. Finally, nodules may contribute more to meiofauna diversity at larger spatial scales. In the GSR contract area, variability in nematode taxonomic composition, indicative of beta or turnover diversity, was higher between nodule crevice than between sediment samples.

### Recommendations for environmental management

Contractors are obliged to collect environmental baseline data within their contract area(s)^59^. This data should also inform the environmental impact assessment (EIA) which is required to obtain an exploitation license^60^. This EIA should guide effective environmental management of individual contract areas. One contract area-scale environmental management tool is the designation of preservation and impact reference zones (PRZs and IRZs). Their main objective is to monitor mining impacts, although they could also play a conservational role^24^. Moreover, other set-aside areas could be established. The CCFZ regional environmental management plan^61^ includes an “Areas of Particular Environmental Interest” (APEIs) network representative of the region’s biodiversity and ecosystem functions, which are to be safeguarded from mining^62^. Several recommendations, from a meiofauna perspective, for environmental management at the contract area and regional scale follow from our study:

- the current sampling effort was insufficient to accurately assess copepod species diversity and to conduct all statistical analyses with sufficient power. We recommend that replication in baseline and consequently monitoring studies should be augmented (and at least doubled for copepod species diversity) to obtain reliable measures of meiofaunal community attributes.
- To meet their primary objectives, PRZs (monitoring at the scale of individual contract areas) and APEIs (conservation at the regional scale) need to be ecologically similar to planned mining areas. The delineation of additional APEIs^63^ and PRZs^24^ is imminent. Since nodule crevices contain meiofauna and nodule size relates to crevice meiofauna abundance, our research strongly supports previous recommendations that PRZs and APEIs should encompass the entire size spectrum of nodules found in the planned mining areas^6,24,64^.
- Since there was no consistent or strong effect of nodule presence on sediment meiofaunal community composition or diversity, nodule-free areas (if unaffected by mining) may help to protect sediment meiofaunal diversity (if connected to other unimpacted populations) and potentially serve as a recruitment source for recolonization of mined nodule-rich areas.
- Artificial substrates can potentially facilitate recolonization by nodule epifauna^65^, yet their success and the exact prerequisites (e.g. metal content, surface texture) remain to be tested in (nodule-bearing) abyssal basins^66^. For the recolonization of crevice meiofauna, the internal structure of crevice networks within these artificial substrates should probably match natural nodules as closely as possible. As for nodule epifauna, the importance of other factors like metal content warrants investigation.

## Materials and methods

### Study area and sampling design

The Global Sea Mineral Resources (GSR) contract area in the northeastern Clarion Clipperton Fracture Zone (CCFZ; northeast Pacific; Fig. 1a) was sampled as part of the environmental baseline characterization. During the GSR exploration expedition in September–October 2015 aboard the MV *Mt Mitchell* (“GSRNOD15A”), three nodule-rich (average nodule abundance > 19 kg m^−2^, see Table 2 in De Smet et al.^67^) sites were sampled: two in zone B4 (B4S03 and B4N01) and one in B6 (B6S02) (Fig. 1b). Sedimentary environmental characteristics of these sites were described by Pape et al.^55^. Based on multibeam backscatter and seabed imagery we identified two potential nodule-rich (“NodRich_A” and “NodRich_B”) and one nodule-free (“NodFree”) station at B4S03, which were sampled in May–June 2017 (“GSRNOD17”) (Fig. 1c). Boxcore samples confirmed the significantly lower nodule abundance at NodFree (0.53 ± 0.72 kg m^−2^) compared to the nodule-rich (NodRich_A: 24.17 ± 1.54 kg m^−2^, NodRich_B: 20.01 ± 5.56 kg m^−2^) stations (see Pasotti et al.^11^). More details on the environmental characterization of these stations are given by Pasotti et al.^11^.

### Sampling strategy and onboard sample processing

#### Nodule-free *vs*. nodule-rich sediments

For the comparison between nodule-free and nodule-rich sediments, 3–4 replicate multicorer (MUC) deployments (internal core diameter: 100 mm) were performed at NodFree and the two nodule-rich stations (NodRich_A and NodRich_B) at site B4S03 during the 2017 expedition aboard MV *Topaz Captain*. The top 0–5 cm (including the 32 µm sieve residue from the overlying bottom water) of one sediment core per deployment was preserved in 10% seawater-buffered formaldehyde for meiofauna community analyses. Two additional cores were analyzed for sedimentary environmental characteristics, including chlorophyll *a* concentrations, organic carbon and nitrogen content and granulometry (see Pasotti et al.^11^ for details on sample processing).

#### Nodule crevices *vs*. sediments

Nodules were collected during two GSR expeditions. During GSRNOD15A, nodules were obtained with the boxcorer (50 × 50 cm) from sites B4S03, B6S02 and B4N01 (Table 1). The B4S03 samples collected in 2015 all originated from station NodRich_A. Analyses were done for three replicate boxcore deployments per site; per boxcore, five nodules were randomly selected. Sediment meiofauna from these sites was sampled in triplicate with a MUC; the resulting data have been published^55^, but are compared here with the nodule crevice meiofauna results. In 2017 (GSRNOD17), we collected the nodules retained within the MUC cores from which the sediment was analyzed for meio- and nematofauna (see previous paragraph). This approach allowed for a comparison between the nodule crevices and the immediately surrounding sediments within these cores. Nodules were carefully rinsed with cold (4 °C) filtered seawater and preserved in 10% seawater-buffered formaldehyde. GSRNOD15A and GSRNOD17 sediment samples were processed similarly. Eight GSRNOD17 MUC cores contained (1 or 2) nodules, but because two nodules broke during transport, we analyzed data from six cores only.

**Table 1:** Positions and depths of samples collected for this study in the GSR contract area.

Expedition	Site	Station	Deployment	Lat	Long	Water depth (m)
GSRNOD15A	B6S02	–	BC011	13.894	− 123.297	4549
			BC013	13.888	− 123.289	4560
			BC015	13.883	− 123.282	4560
	B4S03	NodRich_A	BC018	14.112	− 125.871	4501
			BC019	14.118	− 125.880	4488
			BC021	14.104	− 125.878	4477
	B4N01	–	BC026	14.706	− 125.461	4509
			BC027	14.706	− 125.442	4501
			BC029	14.706	− 125.452	4504
GSRNOD17	B4S03	NodFree	MUC011	14.067	− 125.929	4649
			MUC012	14.059	− 125.921	4575
			**MUC013**	14.054	− 125.924	4573
			MUC020	14.050	− 125.922	4557
		NodRich_A	MUC014	14.036	− 125.925	4537
			**MUC015**	14.029	− 125.925	4555
			**MUC016**	14.034	− 125.929	4545
			**MUC021**	14.036	− 125.910	4550
		NodRich_B	**MUC017**	14.004	− 125.878	4480
			MUC018	14.112	− 125.872	4510
			**MUC019**	14.118	− 125.879	4500

Only at site B4S03 different stations were delineated. Coordinates (lat, long) are expressed in decimal degrees. *BC* boxcorer, *MUC* multicorer. Boxcores were sampled for nodule crevice meiofauna, whilst MUCs were sampled for sediment meiofauna. MUCs denoted in bold were investigated for both sediment and nodule crevice meiofauna.

### Sample analyses

#### Nodules

The dimensions (length, width and height), weight and volume (replacement volume in water) of all polymetallic nodules were measured. The surface of the nodules was carefully rinsed over a 1 mm and 32 µm sieve, and sieve residues were preserved in 4% buffered formaldehyde. These nodule surface samples were not considered further, as we believed we could not be absolutely certain that the meiofauna found in these samples inhabited the nodule surface (“nodule epifauna”) and not the immediately surrounding sediments (“sediment endofauna”), especially for the lower part of the nodules which is embedded in the soft sediments. Next, nodules were fragmented with a hammer, washed over the same set of sieves and residues were fixed in 4% buffered formaldehyde.

#### Meiofauna

Meiofauna was extracted from sediment and nodule sieve residues following Pape et al.^55^. For the sediment samples, the two taxa which typically dominate the meiofauna in (nodule-bearing) abyssal sediments world-wide, i.e. nematodes and copepods, were identified to lower taxonomical level (nematodes: genus, copepods: (morpho)species). The nematode genera, which are hard to distinguish morphologically, especially when dealing with juveniles (e.g., *Thalassomonhystera/Monhystrella*, and *Microlaimus/Aponema*), were placed in so-called genus groups. One nematode genus, *Halalaimus*, was identified to species level using the original descriptions on NeMys^68^. For the nematodes 120 specimens were hand-picked randomly for identification, whilst for the copepods all specimens were considered. Copepod identification was limited to adult harpacticoids because most of the copepodids cannot be identified reliably^69^. For the nodule crevice samples, only nematodes were identified further. Because of the much lower abundance compared to the sediments (median abundance of 530 and 20 individuals in sediments and nodule crevices, respectively) all nematodes in the nodule crevice samples were identified. Nematodes were assigned to trophic groups based on buccal morphology following Wieser^48^: 1A (no or very small toothless buccal cavity), 1B (larger, toothless buccal cavity), 2A (small-medium buccal cavity with small tooth or teeth) and 2B (large buccal cavity with large teeth or mandibles). The relative numerical importance of these trophic groups was examined for the different stations and substrates by comparing trophic composition between stations and substrates. Buccal morphology proved to be indicative of feeding behavior in shallow marine waters^70^, though more recent studies showed the link to be ambiguous^50,51,71^ and thus resulting data need to be interpreted with care. Data from three nodules, which got fractured during sample transport, were omitted from the analyses, as the distinction between nodule crevice (“nodule endofauna”) and surface meiofauna (“nodule epifauna”) was then impossible. The final dataset comprised 51 nodules.

### Data analysis

Except for the BIO-ENV BVStep analysis, which was done in Primer v6 (to date there is no unbiased similar statistical test available in R)^72^, all data analyses were run in R^73^. The significance level was set at 0.05; P-values were not corrected for multiple testing. Permutational tests were done for 9999 permutations or all possible. All plots, except for the UpSet plots (see further), were created using packages “ggplot2”^74^ and “cowplot”^75^. Data are presented as means ± SD, unless indicated otherwise. For all statistical tests employed to investigate differences in abundance, composition or diversity, between stations or substrates, the null hypothesis was that there were no differences.

#### Nodule-free *vs*. nodule-rich sediments

Meiofauna abundance was compared between the nodule-free and the two nodule-rich stations (factor Station with levels “NodFree”, “NodRich_A” and “NodRich_B”) using a one-way univariate PERMANOVA analysis (*adonis2* from “vegan”^76^). This test was repeated with sediment volume as a covariate to check for differences between stations given sediment volume differences. Potential relationships between meiofauna abundance and the environmental variables measured (i.e. sediment chlorophyll *a*, total organic carbon and nitrogen content, porosity, median grain size, content of sand, silt and clay, and the sorting coefficient) were examined by Spearman Rank correlation tests. Differences in meiofauna (higher taxa), copepod (families and (morpho)species) and nematode (families and genera, *Halalaimus* species and trophic groups) community composition between stations were tested using a multivariate one-way PERMANOVA (factor Station) and visualized using Principal Coordinated Analysis (PcoA) plots. Because MUC014 contained a large nodule and therefore virtually no sedimentary meiofauna, this core was excluded from community analyses. Following significant main PERMANOVA tests, a PERMDISP analysis (*betadisper* followed by *permutest* in “vegan”) was executed to check whether multivariate dispersions were homogeneous. In case of heterogeneous dispersions, PCoA plots were examined to discern whether this dispersion effect was accompanied by a location effect (i.e. a shift in multivariate space)^43^. Where appropriate, pairwise PERMANOVA tests, using “pairwiseAdonis”^77^, were conducted. For meiofauna abundances, Euclidean distances were used to construct dissimilarity matrices. Multivariate biological data (i.e. taxonomic and trophic composition) were Hellinger transformed (to down-weigh the importance of dominant taxa or groups and to account for different total abundances) before constructing Bray–Curtis dissimilarity matrices. A BVStep analysis (Spearman Rank correlation) was performed to relate taxonomic composition to the environmental data (0–5 cm sediment depth). A simper analysis, from “vegan”, showed which taxa were most responsible for differences between stations.

Nematode and copepod diversity were compared between stations by constructing rarefaction/extrapolation (R/E) plots based on sample size (number of individuals) and sample coverage for Hill numbers of order q = 0 (taxon richness), 1 (Shannon diversity) and 2 (Simpson diversity)^78^ using the “iNEXT” package^79^. Plots are constructed by both interpolating (rarefying to smaller sample sizes) and extrapolating (predicting, guided by asymptotic estimators) Hill numbers^78^. The higher the order of q, the less sensitive the Hill number is to sample size and rare taxa. The base sample size and coverage, which is the maximal sample size and coverage, respectively, for which assemblages can be reliably compared, were indicated. Non-overlapping 95% confidence intervals were regarded as evidence for significant differences; intersecting intervals, however, did not necessarily indicate insignificant dissimilarities^80^. Taxon distribution across stations was examined with UpSet plots for nematode genera, *Halalaimus* species and copepod species using “UpSetR”^81^.

Whenever differences in meiobenthic community attributes between the two nodule-rich stations were consistently smaller than those between both nodule-rich stations and the nodule-free station, this was considered a potential effect of nodule presence.

#### Nodule crevices *vs*. sediments

Relationships between nodule dimensions and nodule crevice meiofauna abundance were analyzed using Spearman Rank correlations. The presence of meiofauna and nematode taxa in the two substrates, i.e. sediments and nodule crevices, was investigated for the combined GSRNOD15A and GSRNOD17 dataset with UpSet plots. Meiofauna higher taxon and nematode genus composition (after Hellinger transformation and the generation of a Bray–Curtis dissimilarity matrix), as well as nematode trophic composition, was compared between sediments and nodule crevices using PERMANOVA and visualized through PcoA. Again, significant PERMANOVA tests were followed by PERMDISP and simper analyses. Because of the differential sampling strategy during GSRNOD15A and GSRNOD17, the resulting meio- and nematofauna community datasets were analyzed with a different design. Owing to the much lower number of *Halalaimus* specimens inside the nodules compared to the sediments, we did not compare *Halalaimus* species diversity or composition between the two substrates either graphically or statistically.

For the nodules sampled with the boxcorer during GSRNOD15A, the median (less sensitive to outliers than the mean) of the meiofauna data from the five nodules was first calculated per boxcore. This allowed for a more straightforward comparison with the sediment data, with three replicate samples each for the nodule crevice (boxcores) and the sediment meiofauna (MUCs). This dataset was then subjected to a two-way PERMANOVA with factors Site (levels: “B6S02”, “B4S03” and “B4N01”) and Substrate (levels: “sediment” and “nodule crevice”). For the nodules sampled with the MUC during GSRNOD17, a one-way PERMANOVA was run with Substrate as a factor (levels: “sediment” and “nodule crevice”), and MUC cores set as strata to account for the dependency between nodules and sediments from the same MUC core (hereby blocking the permutations per core). The same permutation design, specified with the package “permute”^82^, was used in the simper analyses. Given the high variability in counts between nodules, median relative abundances were reported.

Nematode genus diversity was compared between nodule crevices and sediments using R/E plots in “iNEXT”. Additionally, nematode genus beta diversity (based on presence-absences) within these cores (i.e. between the nodule crevices and the sediment) was partitioned in a turnover (replacement of taxa) and nestedness (loss or gain of taxa) component using “betapart”^83^.

#### Nodule crevices *plus* sediments

The contribution of crevice meio- and nematofauna to total meiofauna and nematode diversity was determined for the GSRNOD17 MUC cores by comparing nodule crevice with total (sediment + nodule crevice) diversity (for Hill numbers of orders q = 0, 1 and 2) for the same base sample size in “iNEXT”.

## Supplementary Information

Supplementary Information.

## Data Availability

All raw data were submitted to the DeepData database of the International Seabed Authority (https://data.isa.org.jm/isa/map/).
