# Supplementary material for: Potential impacts of polymetallic nodule removal on deep-sea meiofauna
Source: Sci Rep. 2021 Oct 7;11:19996. doi: 10.1038/s41598-021-99441-3 (PMC8497503; doi:10.1038/s41598-021-99441-3)
Supplement: Supplementary file 1 — Supplementary Information. [file 41598_2021_99441_MOESM1_ESM.docx]

Supplementary material


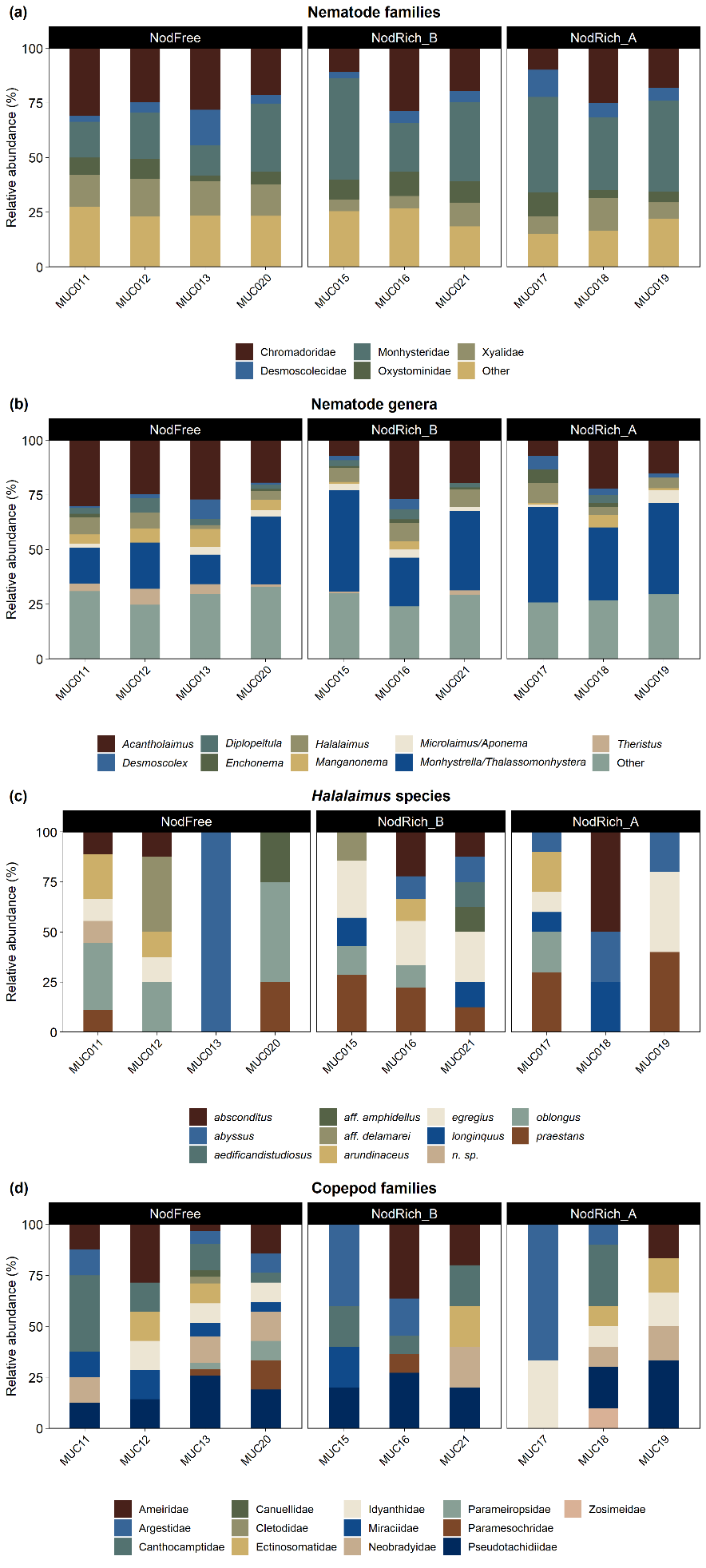


**Figure S1.** **Barplots showing taxonomic composition at the nodule-free (NodFree) and nodule-rich (NodRich_A and NodRich_B) stations**. **(a)** nematode families, **(b)** nematode genera, **(c)** Halalaimus species and **(d)** copepod families Plot (a) and (b) show the most abundant nematode families (≥10% relative abundance in at least one of the MUC cores) and genera (≥5% of relative abundance in at least one of the MUC cores), respectively.


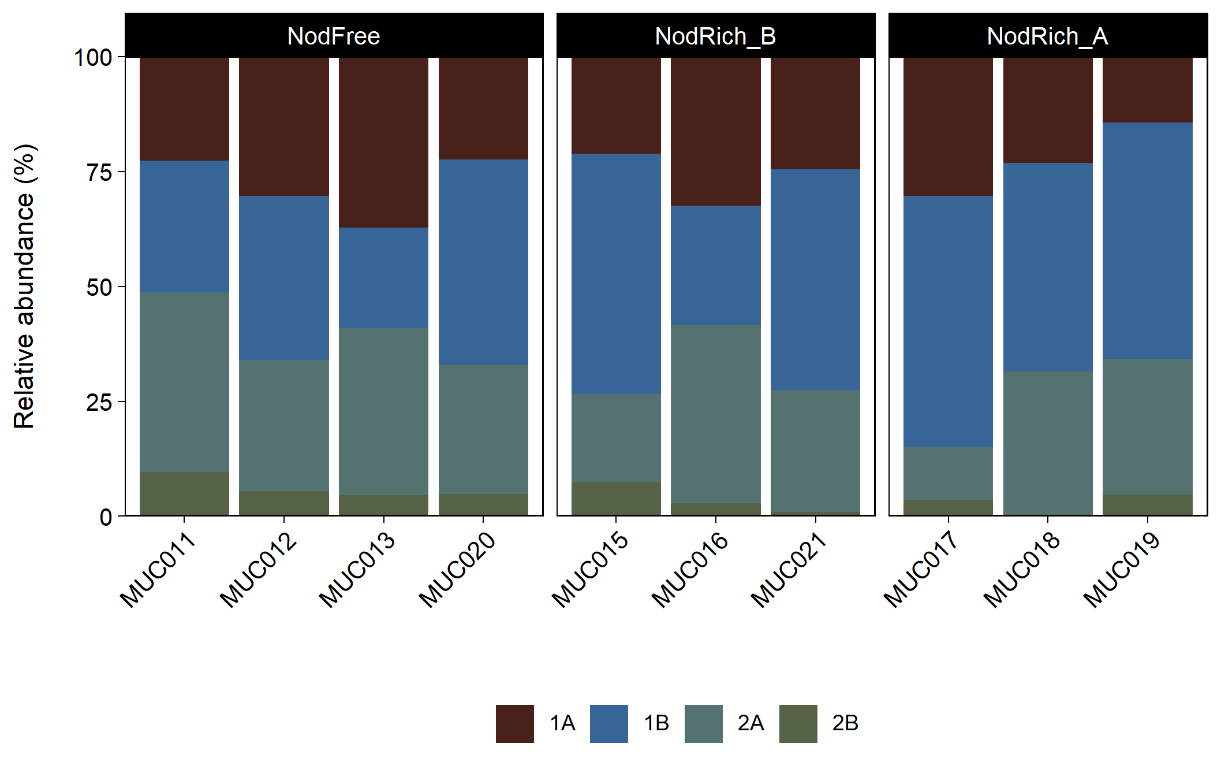


**Figure S2. Barplot showing nematode trophic** **composition at the nodule-free (NodFree) and nodule-rich (NodRich_A and NodRich_B) stations**. 1A: no or very small toothless buccal cavity, 1B: larger toothless buccal cavity, 2A: small-medium buccal cavity with small tooth or teeth and 2B: large buccal cavity with large teeth or mandibles.


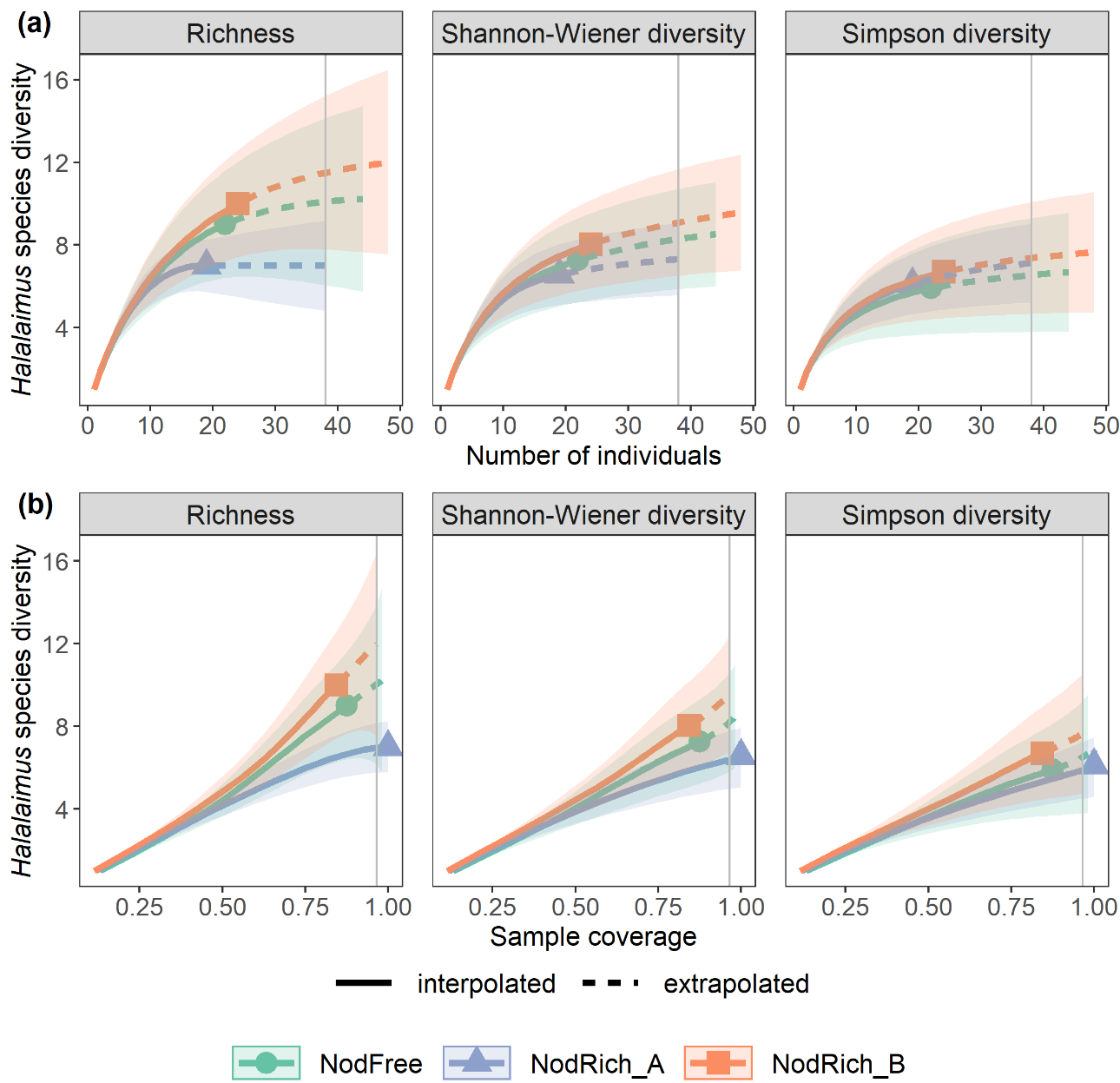


**Figure S3.** **Halalaimus species diversity at the** **nodule-free (NodFree) and nodule-rich (NodRich_A and NodRich_B) stations.** Shown are **(a)** sample-size and **(b)** sample coverage-based rarefaction (interpolated) and extrapolation (predicted, guided by asymptotic estimators) curves based on abundances. The different panels show Hill numbers of orders (q) 0 (richness), 1 (Shannon-Wiener diversity) and 2 (Simpson diversity). The vertical grey line denotes in (a) the base sample size and in (b) the base sample coverage. Shaded areas represent 95% confidence intervals.


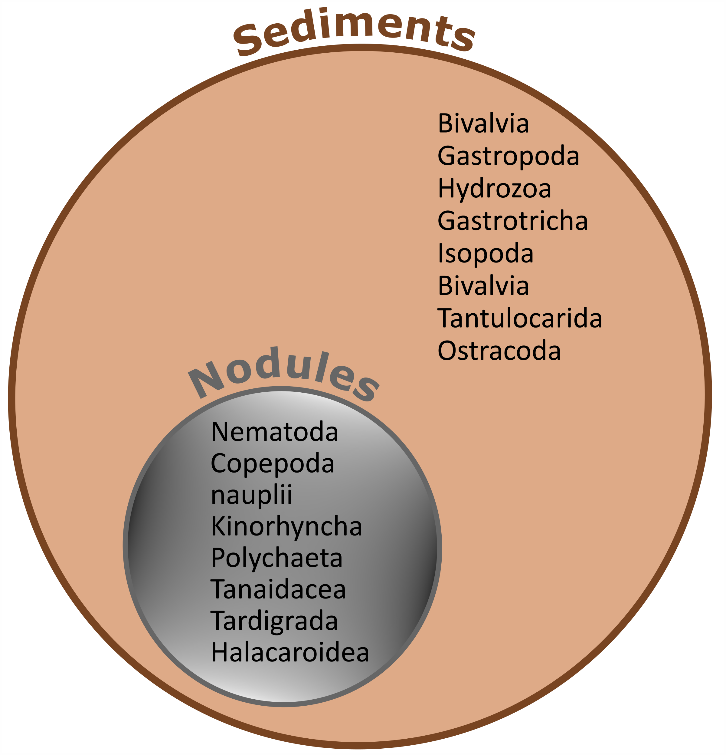


**Figure S4. Venn diagram showing the higher meiofauna taxa present in the sediments and nodule crevices.** As there were no taxa restricted to the nodule crevices, there is no intersection between Nodules and Sediments in the venn diagram.


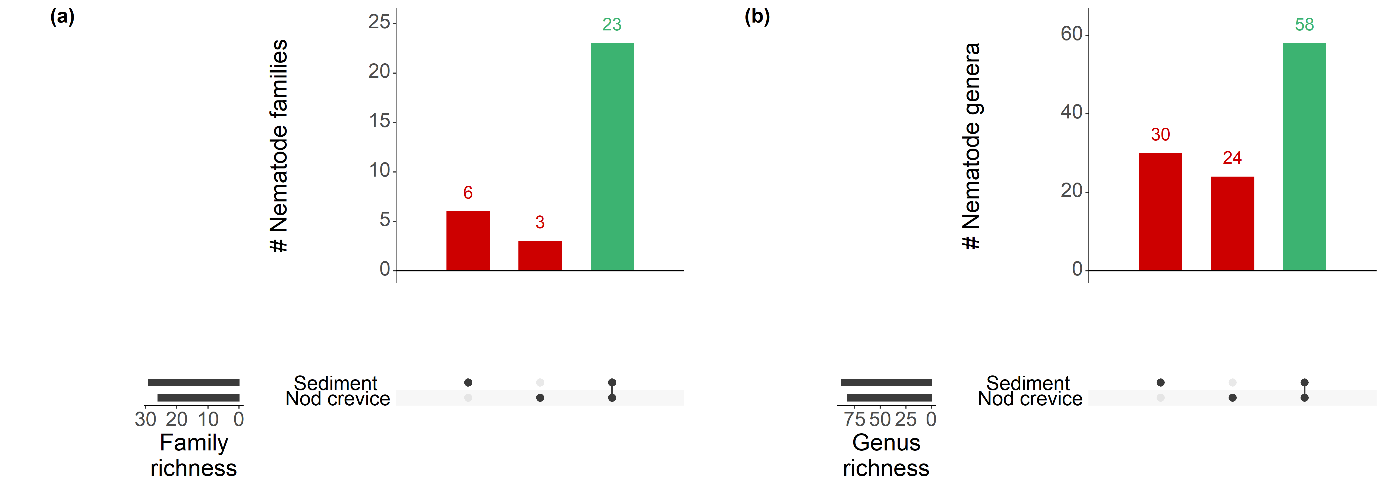


**Figure S5.** **UpSet plots for nematode taxa for nodule (Nod) crevice and sediment samples, showing the number of unique and shared taxa.** **(a)** Families and **(b)** genera. The color of the bars denotes the commonness of taxa, with red: unique taxa and green: taxa shared between substrates.


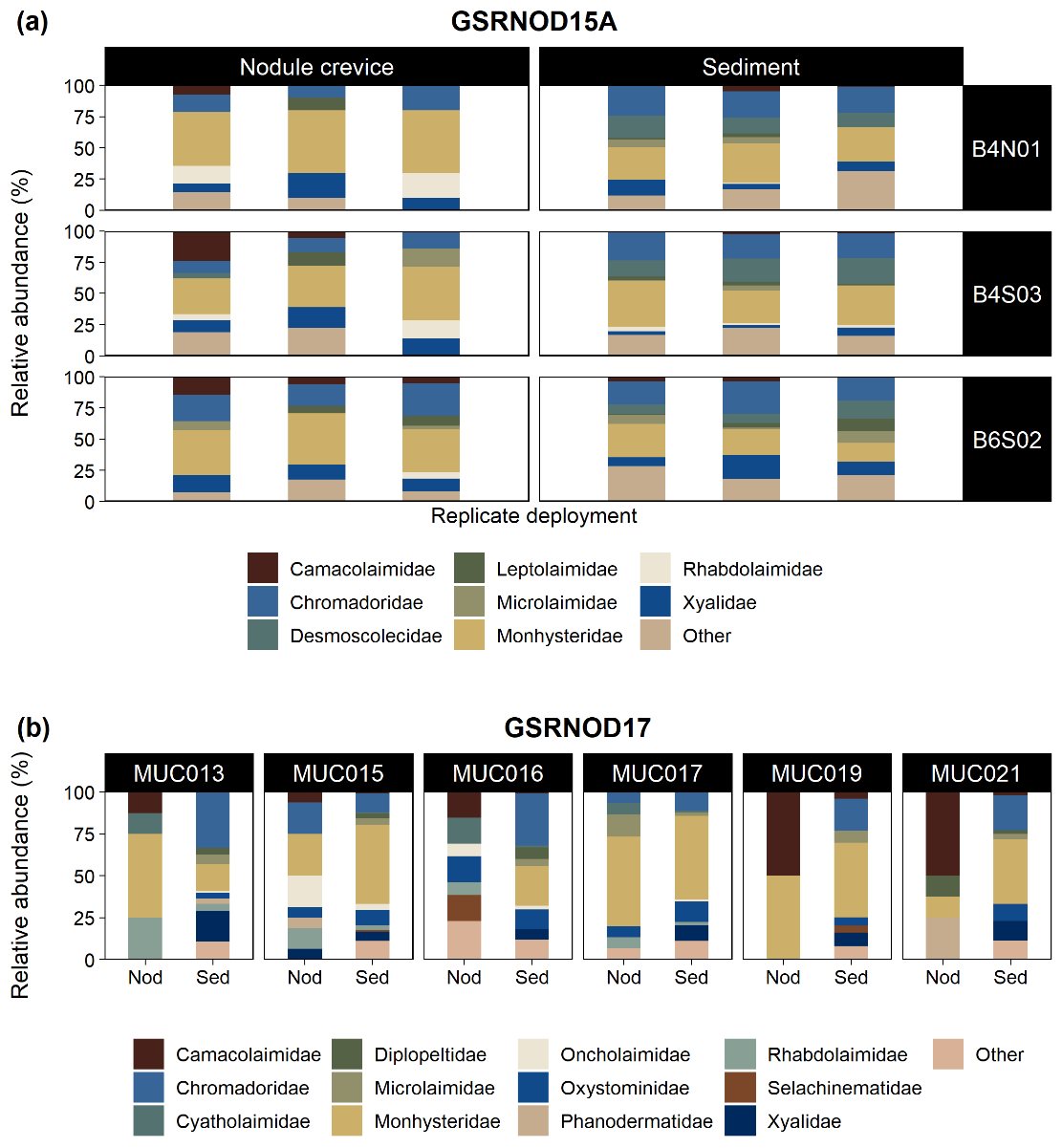


**Figure S6.** Nematode family composition for **(a)** GSRNOD15A and **(b)** GSRNOD17 nodule crevice (nod) and sediment (sed) samples. Colors denote dominant families, having a relative abundance of ≥10% in at least one sample


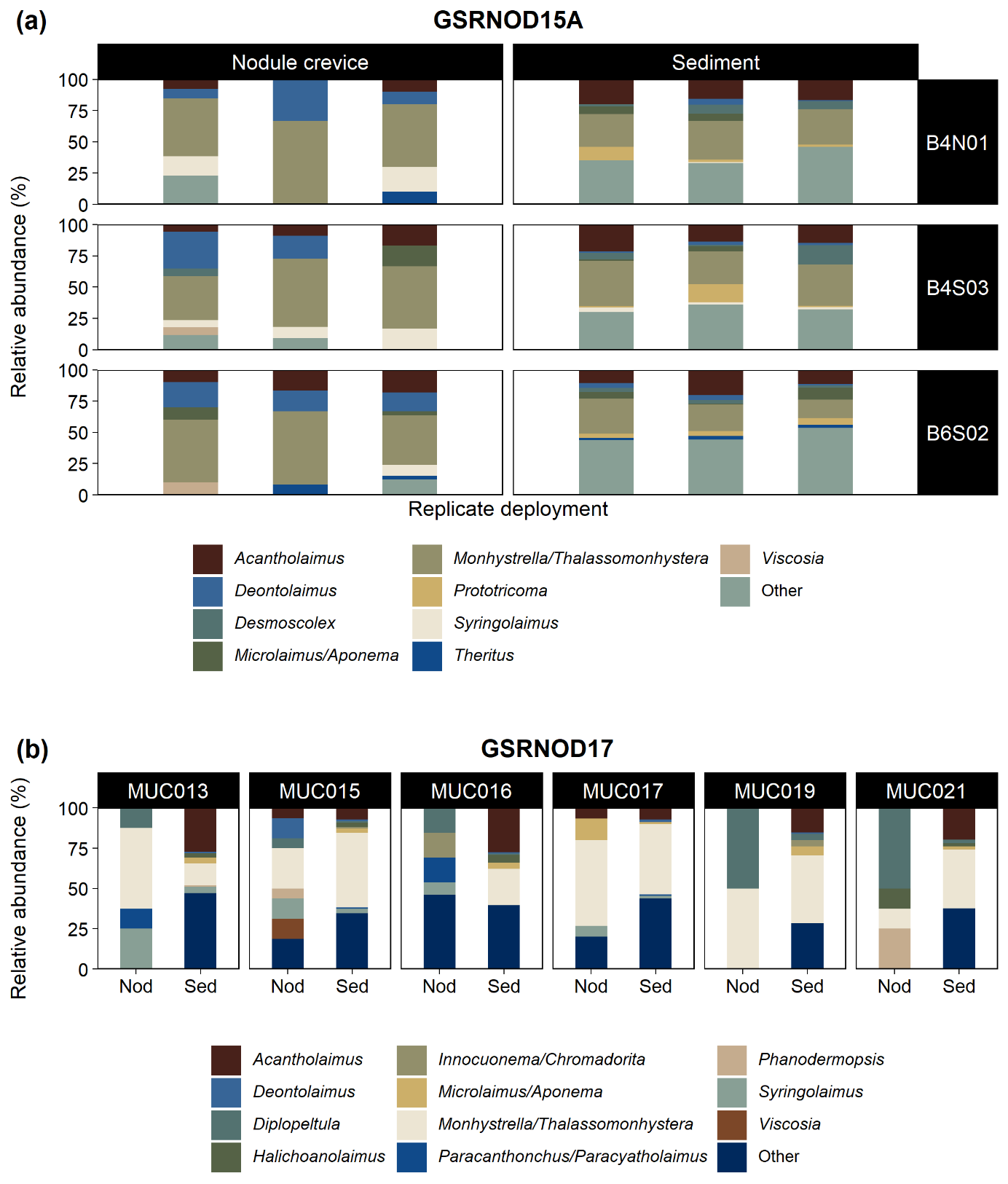


**Figure S7.** Nematode genus composition for **(a)** GSRNOD15A and **(b)** GSRNOD17 nodule crevice (nod) and sediment (sed) samples. Colours denote dominant genera or genus groups, having a relative abundance of ≥10% in at least one of the nodule crevice or sediment samples.


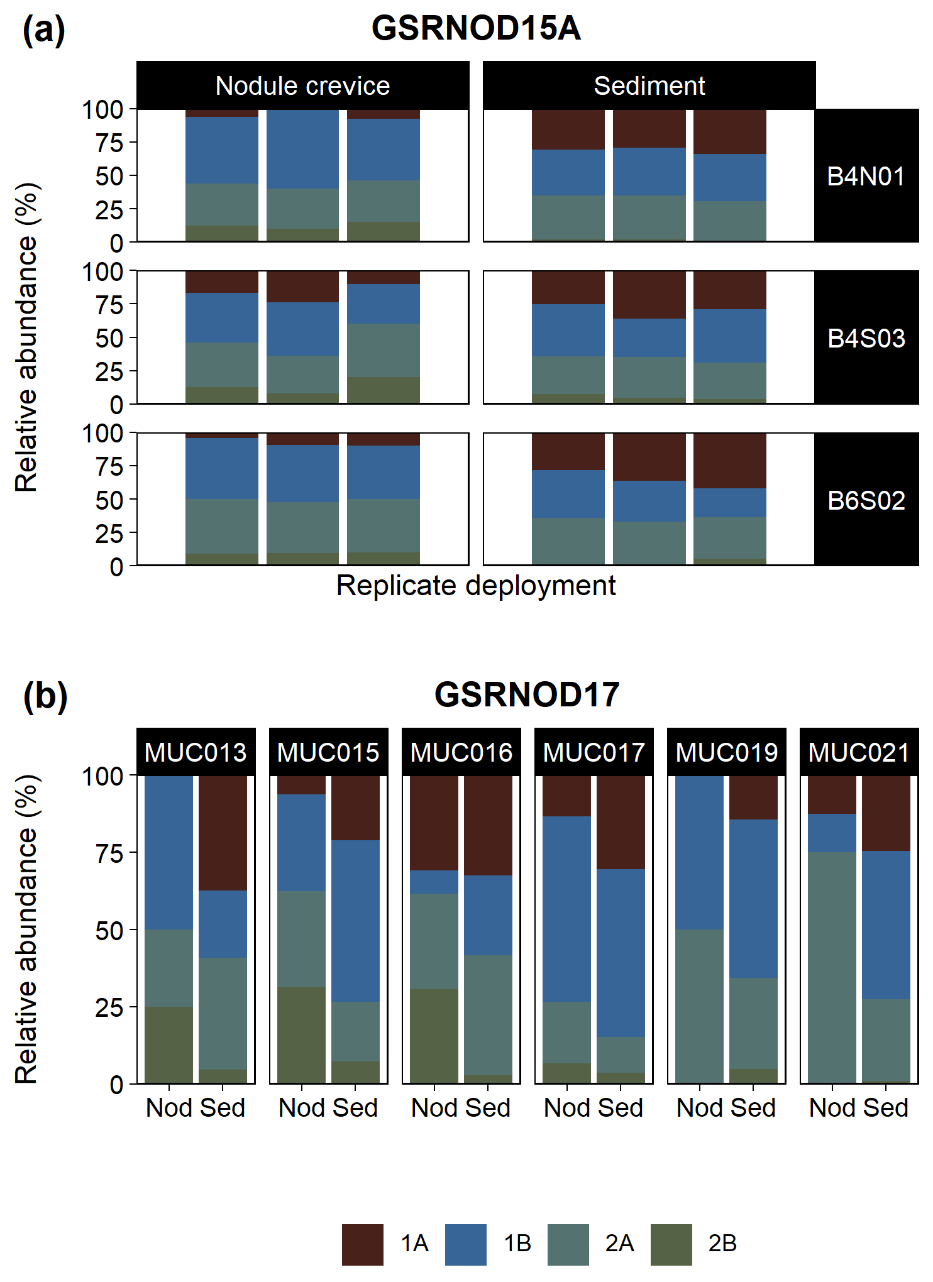


**Figure S8.** Nematode trophic composition for **(a)** GSRNOD15A and **(b)** GSRNOD17 nodule crevice (nod) and sediment (sed) samples. 1A: no or very small toothless buccal cavity, 1B: larger toothless buccal cavity, 2A: small-medium buccal cavity with small tooth or teeth and 2B: large buccal cavity with large teeth or mandibles.

| Taxon composition | Best matching sedimentary environmental variables | R (P) |
| --- | --- | --- |
| Meiobenthic higher taxa | Clay | 0.44 (0.22) |
| Nematode families | Chl a, Porosity, Silt | 0.24 (0.72) |
| Nematode genera | TN | 0.20 (0.80) |
| *Halalaimus* species | Porosity | 0.18 (0.80) |
| Copepod families | TOC, Silt | 0.39 (0.36) |
| Copepod species | TN, Silt | 0.33 (0.45) |

**Table S1. Results of the BVSTEP analyses matching taxon composition with the sedimentary environmental variables measured**. R=Spearman-Rank correlation coefficient. Chl a=chlorophyll a (µg g^-1^), TOC=total organic carbon content (%), TN = total nitrogen content (%)
